# Estimating Non-Gaussianity of a Quantum State by Measuring Orthogonal Quadratures

**DOI:** 10.3390/e24020289

**Published:** 2022-02-18

**Authors:** Jiyong Park

**Affiliations:** School of Basic Sciences, Hanbat National University, Daejeon 34158, Korea; jiyong.park@hanbat.ac.kr

**Keywords:** non-Gaussianity, relative entropy, homodyne detection

## Abstract

We derive the lower bounds for a non-Gaussianity measure based on quantum relative entropy (QRE). Our approach draws on the observation that the QRE-based non-Gaussianity measure of a single-mode quantum state is lower bounded by a function of the negentropies for quadrature distributions with maximum and minimum variances. We demonstrate that the lower bound can outperform the previously proposed bound by the negentropy of a quadrature distribution. Furthermore, we extend our method to establish lower bounds for the QRE-based non-Gaussianity measure of a multimode quantum state that can be measured by homodyne detection, with or without leveraging a Gaussian unitary operation. Finally, we explore how our lower bound finds application in non-Gaussian entanglement detection.

## 1. Introduction

Non-Gaussian quantum resources, such as non-Gaussian states and operations, are indispensable in continuous-variable (CV) quantum information [1,2] because their Gaussian counterparts have fundamental limitations in CV quantum information tasks. For instance, there are Gaussian no-go theorems for quantum entanglement distillation [3,4,5], quantum error correction [6], and quantum bit commitment [7]. Furthermore, non-Gaussian quantum resources can be advantageous over Gaussian quantum resources. Non-Gaussian states can be more noise-resilient than Gaussian states in optical nonclassicality [8] and quantum entanglement [9,10,11,12,13]. Non-Gaussian operations can improve nonclassical properties including optical nonclassicality [14,15,16], quantum entanglement [17,18,19,20,21,22,23,24,25,26], and quantum nonlocality [26,27,28,29]. Moreover, they can enhance the performance of CV quantum information protocols, such as quantum teleportation [30,31,32,33,34], quantum linear amplification [35,36,37], quantum dense coding [38], quantum key distribution [39], and quantum target detection [40].

Probing non-Gaussianity in CV quantum information, it is essential to obtain a faithful quantifier for non-Gaussianity. Thus, non-Gaussianity measures for quantum states have been proposed by employing the quantum Hilbert–Schmidt distance [41], quantum relative entropy (QRE) [42], Wehrl entropy [43], quantum Rényi relative entropy [44], Wigner–Yanase skew information [45], and Kullback–Leibler divergence (KLD) [46]. Furthermore, quantum non-Gaussianity, i.e., a stronger form of non-Gaussianity, has been introduced to distinguish genuinely non-Gaussian states from classical mixtures of Gaussian states [47]. The quantum non-Gaussianity measures have been proposed by using the Wigner logarithmic negativity [48,49], quantum relative entropy [50], stellar representation [51], and robustness [52]. Additionally, quantum non-Gaussianity witnesses have been theoretically proposed [47,53,54,55,56,57,58,59,60,61,62,63,64,65] and experimentally demonstrated [62,63,64,65,66,67] to certify quantum non-Gaussianity efficiently. Here, our main interest is in non-Gaussianity measures that characterize the difference between a quantum state and its reference Gaussian state. Although the non-Gaussianity measures are helpful in characterizing non-Gaussian quantum resources, resource-intensive quantum state tomography [68] is generally required to obtain the exact value of the measures in general. For the case of QRE-based non-Gaussianity measure, observable lower bounds have been developed to address this issue by using the information of the covariance matrix in conjunction with the photon number distribution [69] and the negentropy of a quadrature distribution [46]. The former, i.e., the lower bound in [69], works better than the latter, i.e., the lower bound in [46], especially for quantum states with rotational symmetry in phase space but demands two measurement setups, i.e., homodyne detection and photon-number-resolving detection, in general. If there is a priori information that the quantum state has rotational symmetry in phase space, the lower bound in [69] can be deduced from a quadrature distribution. By contrast, the latter requires only homodyne detection always. In addition, the latter can be used to detect non-Gaussian entanglement in conjunction with partial transposition [46], which may be impossible for the former. Here, we investigate whether an improved lower bound can be obtained by exploiting the negentropy of more than one quadrature distribution, which may open the way to detect non-Gaussian entanglement untestable by the method proposed in [46].

In this study, we show that the sum of the negentropies for two quadrature distributions with the maximum and minimum variances provides a lower bound for the QRE-based non-Gaussianity measure of a single-mode quantum state. We demonstrate that our lower bound can be greater than the maximum negentropy of quadrature distributions, i.e., the lower bound proposed in [46]. We also extend our method to estimate the non-Gaussianity of multimode quantum states with or without the help of a Gaussian unitary operation. We finally propose a method to detect non-Gaussian entangled states beyond the Gaussian positive partial transposition (PPT) entanglement criteria with our lower bound.

## 2. Non-Gaussianity Measures

Here, we briefly discuss the non-Gaussianity measures based on QRE [42] and KLD [46].

The non-Gaussianity measure of a quantum state ρ using QRE was introduced in [42] as

(1) $$N\left(\rho \right)=S(\rho ||\rho_{G})=S\left(\rho_{G}\right)-S\left(\rho \right),$$

where $S(\omega ||\omega^{\prime })=\mathrm{tr}\left(\omega \ln\omega \right)-\mathrm{tr}\left(\omega \ln\omega^{\prime }\right)$ denotes the quantum relative entropy of ω with respect to $\omega^{\prime }$, S(ω)=−tr(ωlnω) denotes the von Neumann entropy of ω, and $\rho_{G}$ denotes the reference Gaussian state of ρ with the same first- and second-order quadrature moments. The first order quadrature moments of an *N*-mode quantum state ρ are given by the expectation value of 2N quadrature operators as $\langle \hat{Q}\rangle ={\left\{\langle {\hat{q}}_{1}\rangle ,\langle {\hat{p}}_{1}\rangle ,\ldots ,\langle {\hat{q}}_{N}\rangle ,\langle {\hat{p}}_{N}\rangle \right\}}^{T}$ with ${\hat{q}}_{j}=\frac{1}{\sqrt{2}}\left({\hat{a}}_{j}+{\hat{a}}_{j}^{†}\right)$ and ${\hat{p}}_{j}=\frac{i}{\sqrt{2}}\left({\hat{a}}_{j}-{\hat{a}}_{j}^{†}\right)$ being the position and momentum operators, respectively, for the *j*th mode. The covariance matrix Γ of the *N*-mode quantum state ρ is a 2N×2N matrix with elements described by the first- and second-order quadrature moments as follows:

(2) $$\Gamma_{jk}=\frac{1}{2}\langle {\hat{Q}}_{j}{\hat{Q}}_{k}+{\hat{Q}}_{k}{\hat{Q}}_{j}\rangle -\langle {\hat{Q}}_{j}\rangle \langle {\hat{Q}}_{k}\rangle ,$$

with j,k∈{1,2,…,2N−1,2N}. Note that an *N*-mode Gaussian state $\rho_{G}$ is uniquely determined by its first-order quadrature moments and the covariance matrix [2]. Furthermore, the von Neumann entropy of the Gaussian state $\rho_{G}$ is obtained as follows:

(3) $$S\left(\rho_{G}\right)=\sum_{j=1}^{N}g\left(\Lambda_{j}\right),$$

where $\Lambda_{j}$ is the *j*th symplectic eigenvalue of the covariance matrix Γ and the function g(x) is given by $g\left(x\right)=\left(x+\frac{1}{2}\right)\ln\left(x+\frac{1}{2}\right)-\left(x-\frac{1}{2}\right)\ln\left(x-\frac{1}{2}\right)$ [2]. We also note that Equation (3) can be simplified as

(4) $$S\left(\rho_{G}\right)=g\left(\sqrt{\det\Gamma }\right),$$

when $\rho_{G}$ is a single-mode Gaussian state.

The non-Gaussianity measure of a quantum state ρ by KLD was developed in [46] as

(5) $$N_{\mathrm{KL}}\left(\rho \right)=\max_{\Theta ,\Phi }J_{\rho }\left(Q_{\Theta ,\Phi }\right),$$

where $J_{\rho }\left(Q_{\Theta ,\Phi }\right)$ denotes the negentropy [70] of the probability distribution for an *N*-mode quadrature operator ${\hat{Q}}_{\Theta ,\Phi }=\sum_{j=1}^{N}c_{j}{\hat{q}}_{j,\phi_{j}}$. Here, ${\hat{q}}_{j,\phi_{j}}={\hat{q}}_{j}\cos\phi_{j}+{\hat{p}}_{j}\sin\phi_{j}$ is a rotated quadrature operator for the *j*th mode, $\Phi ={\left(\phi_{1},\phi_{2},\ldots ,\phi_{N}\right)}^{T}$ is the set of rotation angles $\phi_{j}$, and $\Theta ={\left(\theta_{1},\theta_{2},\ldots ,\theta_{N-1}\right)}^{T}$ is the set of angular coordinates that determines the superposition coefficient $c_{j}$ as

(6) $$c_{j}=\left\{\begin{array}{cc} \cos\theta_{1} & for\;j\;=\;1,\\ \cos\theta_{j}\prod_{k=1}^{j-1}\sin\theta_{k} & for\;1\;<\;j\;<\;N,\\ \prod_{k=1}^{N-1}\sin\theta_{k} & for\;j\;=\;N. \end{array}\right.$$

Furthermore, the negentropy J(X) of a probability distribution *X* is given by

(7) $$J\left(X\right)=D_{\mathrm{KL}}\left(X|\right|X_{G}),$$

where $D_{\mathrm{KL}}\left(X||Y\right)=\int d\mu X\left(\mu \right)\left[\ln X\left(\mu \right)-\ln Y\left(\mu \right)\right]$ is the KLD between two probability distributions *X* and *Y* [71], and $X_{G}$ is the reference Gaussian distribution of *X* with the same first- and second-order moments as *X*. Equation (7) can be rewritten as follows:

(8) $$J\left(X\right)=H\left(X_{G}\right)-H\left(X\right),$$

where H(X)=−∫dμX(μ)lnX(μ) denotes the differential entropy of the probability distribution *X* [71].

We have shown in [46] that $N_{\mathrm{KL}}\left(\rho \right)$ in Equation (5) provides a lower bound for N(ρ) in Equation (1) as

(9) $$N\left(\rho \right)\geq N_{\mathrm{KL}}\left(\rho \right),$$

which allows us to estimate N(ρ) by measuring a quadrature distribution:

(10) $$N\left(\rho \right)\geq N_{\mathrm{KL}}\left(\rho \right)\geq J_{\rho }\left(Q_{\Theta ,\Phi }\right).$$

## 3. A Lower Bound for Single-Mode Non-Gaussianity

Here, we show that a function of the negentropies for two quadrature distributions provides a lower bound of the non-Gaussianity N(ρ) for a single-mode quantum state ρ as

(11) $$N\left(\rho \right)\geq N_{\mathrm{LB}}\left(\rho \right)\equiv J_{\rho }\left(Q_{\phi_{S}}\right)+J_{\rho }\left(Q_{\phi_{S}+\frac{\pi }{2}}\right)+\ln\frac{2}{e},$$

where $\phi_{S}$ and $\phi_{S}+\frac{\pi }{2}$ are the phase angles minimizing and maximizing the variance of the quadrature distributions, i.e., $\min_{\phi }\langle \Delta {\hat{q}}_{\phi }^{2}\rangle =\langle \Delta {\hat{q}}_{\phi_{S}}^{2}\rangle$ and $\max_{\phi }\langle \Delta {\hat{q}}_{\phi }^{2}\rangle =\langle \Delta {\hat{q}}_{\phi_{S}+\frac{\pi }{2}}^{2}\rangle$, respectively, with $\langle \Delta {\hat{q}}_{\phi }^{2}\rangle =\langle {\hat{q}}_{\phi }^{2}\rangle -{\langle {\hat{q}}_{\phi }\rangle }^{2}$.

To this aim, we first show that

(12) $$H_{\rho_{G}}\left(Q_{\phi_{S}}\right)+H_{\rho_{G}}\left(Q_{\phi_{S}+\frac{\pi }{2}}\right)=\ln\left(\pi e\right)+S_{2}\left(\rho_{G}\right),$$

where $S_{\alpha }\left(\rho \right)=\frac{1}{1-\alpha }\ln\left(\mathrm{tr}\rho^{\alpha }\right)$ is the quantum Rényi-α entropy of a quantum state ρ, which becomes the von Neumann entropy S(ρ) in the limit of α→1. Every single-mode Gaussian state can be described as a displaced squeezed thermal state:

(13) $$\sigma =\hat{D}\left(\beta \right)\hat{S}\left(r,\varphi \right)\tau_{\bar{n}}{\hat{S}}^{†}\left(r,\varphi \right){\hat{D}}^{†}\left(\beta \right),$$

where $\tau_{\bar{n}}=\sum_{n=0}^{\infty }\frac{{\bar{n}}^{n}}{{\left(\bar{n}+1\right)}^{n+1}}\left|n\rangle \langle n\right|$ is the thermal state with mean photon number $\bar{n}$, $\hat{D}\left(\beta \right)=\exp\left(\beta {\hat{a}}^{†}-\beta^{*}\hat{a}\right)$ is the displacement operator with complex amplitude β, and $\hat{S}\left(r,\varphi \right)=\exp\left[-\frac{r}{2}\left\{e^{2i\varphi }{\left({\hat{a}}^{†}\right)}^{2}-e^{-2i\varphi }{\hat{a}}^{2}\right\}\right]$ is the squeezing operator with squeezing strength *r* and squeezing direction φ. The elements of the covariance matrix for the state σ are given by

(14) $$\begin{array}{cc} \Gamma_{11} & =\left(\bar{n}+\frac{1}{2}\right)\left(\cosh2r-\sinh2r\cos2\varphi \right),\\ \Gamma_{22} & =\left(\bar{n}+\frac{1}{2}\right)\left(\cosh2r+\sinh2r\cos2\varphi \right),\\ \Gamma_{12} & =\Gamma_{21}=-\left(\bar{n}+\frac{1}{2}\right)\sinh2r\sin2\varphi . \end{array}$$

In this case, the variance of the quadrature distribution with the phase angle ϕ is given by

(15) $$\begin{array}{cc} \langle \Delta {\hat{q}}_{\phi }^{2}\rangle & =\Gamma_{11}\cos^{2}\phi +\Gamma_{22}\sin^{2}\phi +2\Gamma_{12}\sin\phi \cos\phi \\ \; & =\left(\bar{n}+\frac{1}{2}\right)\left\{\cosh2r-\sinh2r\cos2\left(\phi -\varphi \right)\right\}, \end{array}$$

which is minimized and maximized at ϕ=φ and $\phi =\varphi +\frac{\pi }{2}$, respectively. Since the differential entropy of a Gaussian distribution with the variance *v* is expressed as $\frac{1}{2}\ln\left(2\pi ev\right)$ [71] and the quantum Rényi-2 entropy of the single-mode Gaussian state σ in Equation (13) is determined by $S_{2}\left(\sigma \right)=\ln\left(1+2\bar{n}\right)$ [72], we obtain

(16) $$\begin{array}{c} H_{\sigma }\left(Q_{\phi_{S}}\right)+H_{\sigma }\left(Q_{\phi_{S}+\frac{\pi }{2}}\right)=\ln\left(\pi e\right)+S_{2}\left(\sigma \right). \end{array}$$

Using the ordering property of the quantum Rényi-α entropy $S_{1}\left(\rho \right)\geq S_{2}\left(\rho \right)$ and the entropic quantum uncertainty relation $H_{\rho }\left(Q_{\phi }\right)+H_{\rho }\left(Q_{\phi +\frac{\pi }{2}}\right)\geq \ln\left(2\pi \right)+S_{1}\left(\rho \right)$ [73] in conjunction with Equation (12), we finally have

(17) $$\begin{array}{cc} N_{\mathrm{LB}}\left(\rho \right) & =J_{\rho }\left(Q_{\phi_{S}}\right)+J_{\rho }\left(Q_{\phi_{S}+\frac{\pi }{2}}\right)+\ln\frac{2}{e}\\ \; & \leq S_{2}\left(\rho_{G}\right)-S_{1}\left(\rho \right)\\ \; & \leq S_{1}\left(\rho_{G}\right)-S_{1}\left(\rho \right)\\ \; & =N(\rho ), \end{array}$$

which proves Equation (11).

It should be noted that $N_{\mathrm{LB}}\left(\rho \right)$ can fail to be positive because of the negative constant, i.e., $\ln\frac{2}{e}\approx -0.307$. For a single-mode quantum state ρ with $N_{\mathrm{KL}}\left(\rho \right)<\ln\frac{e}{2}\approx 0.307$, $N_{\mathrm{KL}}\left(\rho \right)$ is always greater than $N_{\mathrm{LB}}\left(\rho \right)$. Therefore, it is necessary to determine whether $N_{\mathrm{LB}}\left(\rho \right)$ can outperform $N_{\mathrm{KL}}\left(\rho \right)$ or not. Some examples are presented in the following subsections.

### 3.1. Fock States

The quadrature distribution for a Fock state |n〉 is expressed by

(18) $$Q_{\left|n\rangle \langle n\right|}\left(q_{\phi }\right)=\frac{1}{2^{n}n!\sqrt{\pi }}e^{-q_{\phi }^{2}}H_{n}{\left(q_{\phi }\right)}^{2},$$

where $H_{n}\left(x\right)$ is a Hermite polynomial of the order *n* [74]. The covariance matrix of the Fock state is given by a 2×2 diagonal matrix, i.e., $\Gamma =\mathrm{diag}(\frac{1}{2}+n,\frac{1}{2}+n)$, which yields N(|n〉〈n|)=(n+1)ln(n+1)−nlnn.

**Figure 1:**
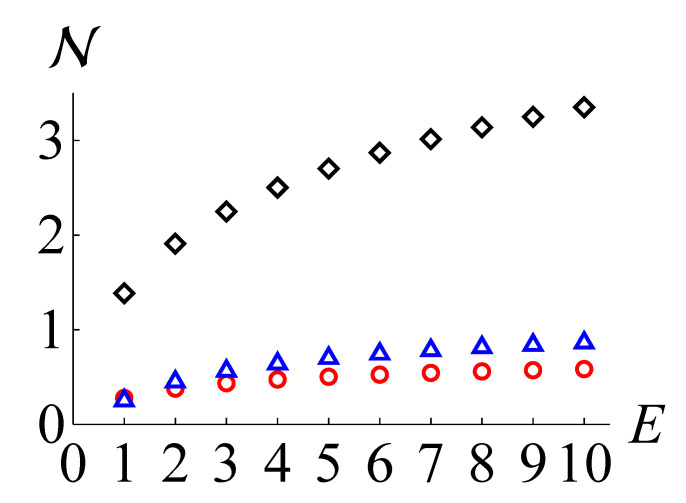
The non-Gaussianity measure based on QRE N(ρ) (black diamond), the maximum negentropy of quadrature distributions $N_{\mathrm{KL}}\left(\rho \right)$ (red circle), and our lower bound $N_{\mathrm{LB}}\left(\rho \right)$ (blue triangle) for Fock states against the mean photon number $E=\mathrm{tr}(\rho {\hat{a}}^{†}\hat{a})$.

In Figure 1, we plot N(ρ), $N_{\mathrm{KL}}\left(\rho \right)$, and $N_{\mathrm{LB}}\left(\rho \right)$ as black diamonds, red circles, and blue triangles, respectively, for the Fock states ρ=|n〉〈n|. It is straightforward to obtain the values of $N_{\mathrm{KL}}\left(\rho \right)$ and $N_{\mathrm{LB}}\left(\rho \right)$, because the Fock states are rotationally symmetric in the phase space. We observe that $N_{\mathrm{LB}}\left(\rho \right)$ exceeds $N_{\mathrm{KL}}\left(\rho \right)$ for n≥2.

### 3.2. Four-Headed Cat States

We now examine a four-headed cat state $|\zeta_{\gamma }\rangle =\sqrt{N_{\gamma }}(|\gamma \rangle +|i\gamma \rangle +|-\gamma \rangle +|-i\gamma \rangle )$ [75], where $|\gamma \rangle =\exp\left(-\frac{{\left|\gamma \right|}^{2}}{2}\right)\sum_{n=0}^{\infty }\frac{\gamma^{n}}{\sqrt{n!}}|n\rangle$ denotes a coherent state with complex amplitude γ and the normalization factor $N_{\gamma }$ is given by

(19) $$N_{\gamma }=\frac{\exp(|\gamma {|}^{2})}{8(\cos|\gamma {|}^{2}+\cosh|\gamma {|}^{2})}.$$

The quadrature distribution for the four-headed cat state is written by

(20) $$Q_{|\zeta_{\gamma }\rangle \langle \zeta_{\gamma }|}\left(q_{\phi }\right)=N_{\gamma }\sum_{j=1}^{4}\sum_{k=1}^{4}Q_{|\gamma_{j}\rangle \langle \gamma_{k}|}\left(q_{\phi }\right),$$

where the expression $Q_{|\gamma_{j}\rangle \langle \gamma_{k}|}\left(q_{\phi }\right)=\langle q_{\phi }|\gamma_{j}\rangle \langle \gamma_{k}|q_{\phi }\rangle$ with ${\hat{q}}_{\phi }|q_{\phi }\rangle =q_{\phi }|q_{\phi }\rangle$ [74] is given by

(21) $$Q_{|\gamma_{j}\rangle \langle \gamma_{k}|}\left(q_{\phi }\right)=\frac{1}{\sqrt{\pi }}\exp\left[-\frac{{\left(\gamma_{k}^{*}e^{i\phi }+\gamma_{j}e^{-i\phi }-\sqrt{2}q_{\phi }\right)}^{2}}{2}-\frac{|\gamma_{j}{|}^{2}+{\left|\gamma_{k}\right|}^{2}}{2}+\gamma_{j}\gamma_{k}^{*}\right],$$

where $\left\{\gamma_{1},\gamma_{2},\gamma_{3},\gamma_{4}\right\}=\left\{\gamma ,i\gamma ,-\gamma ,-i\gamma \right\}$.

The covariance matrix of the four-headed cat state is denoted by a 2×2 diagonal matrix, i.e., $\Gamma =\mathrm{diag}(\frac{1}{2}+m,\frac{1}{2}+m)$, with

(22) $$m={\left|\gamma \right|}^{2}\frac{{\sinh|\gamma |}^{2}-\sin{\left|\gamma \right|}^{2}}{{\cosh|\gamma |}^{2}+\cos{\left|\gamma \right|}^{2}},$$

which yields $N(|\zeta_{\gamma }\rangle \langle \zeta_{\gamma }|)=\left(m+1\right)\ln\left(m+1\right)-m\ln m$.

**Figure 2:**
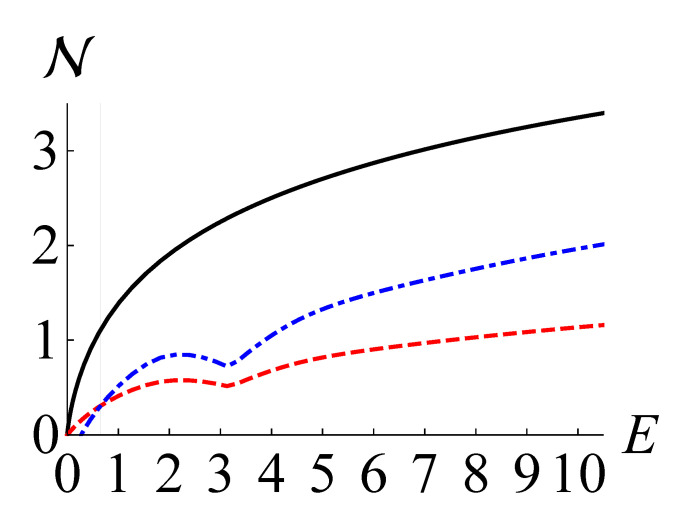
The non-Gaussianity measure based on QRE N(ρ) (black solid), the maximum negentropy of quadrature distributions $N_{\mathrm{KL}}$ (red dashed), and our lower bound $N_{\mathrm{LB}}\left(\rho \right)$ (blue dot-dashed) for four-head cat states against the mean photon number $E=\mathrm{tr}(\rho {\hat{a}}^{†}\hat{a})$.

In Figure 2, we depict N(ρ), $N_{\mathrm{KL}}\left(\rho \right)$, and $N_{\mathrm{LB}}\left(\rho \right)$ as black solid, red dashed, and blue dot-dashed curves, respectively, for the four-headed cat states $\rho =|\zeta_{\gamma }\rangle \langle \zeta_{\gamma }|$. For the blue dot-dashed curves, we have optimized the value of $N_{\mathrm{LB}}\left(\rho \right)$ over the phase angle ϕ, because the variance of the quadrature distribution is the same for all phase angles. We observe that $N_{\mathrm{LB}}\left(\rho \right)$ becomes greater than $N_{\mathrm{KL}}\left(\rho \right)$ for E>0.65 (γ>1.21).

### 3.3. Mixture of Coherent States

We now examine a mixture of coherent states in the form of $\rho =\frac{1}{4}(|\gamma \rangle \langle \gamma |+|i\gamma \rangle \langle i\gamma |+|-\gamma \rangle \langle -\gamma |+|-i\gamma \rangle \langle -i\gamma |)$. Its quadrature distribution is given by

(23) $$Q_{\rho }\left(q_{\phi }\right)=\frac{1}{4}\sum_{k=0}^{3}Q_{|i^{k}\gamma \rangle \langle i^{k}\gamma |}\left(q_{\phi }\right),$$

and its covariance matrix is described by a 2×2 diagonal matrix, i.e., $\Gamma =\mathrm{diag}(\frac{1}{2}+|\gamma {|}^{2},\frac{1}{2}+|\gamma {|}^{2})$. The QRE-based non-Gaussianity measure N(ρ) is obtained by

(24) $$N\left(\rho \right)=g(\frac{1}{2}+{\left|\gamma \right|}^{2})+\sum_{j=1}^{4}\lambda_{j}\log\lambda_{j},$$

where $\lambda_{1}$, $\lambda_{2}$, $\lambda_{3}$, and $\lambda_{4}$ are the eigenvalues of ρ:

(25) $$\begin{array}{cc} \lambda_{1} & =\frac{1}{2}{\exp(-|\gamma |}^{2}{)(\cosh|\gamma |}^{2}+{\cos|\gamma |}^{2}), \end{array}$$

(26) $$\begin{array}{cc} \lambda_{2} & =\frac{1}{2}{\exp(-|\gamma |}^{2}{)(\cosh|\gamma |}^{2}-{\cos|\gamma |}^{2}), \end{array}$$

(27) $$\begin{array}{cc} \lambda_{3} & =\frac{1}{2}{\exp(-|\gamma |}^{2}{)(\sinh|\gamma |}^{2}+{\sin|\gamma |}^{2}), \end{array}$$

(28) $$\begin{array}{cc} \lambda_{4} & =\frac{1}{2}{\exp(-|\gamma |}^{2}{)(\sinh|\gamma |}^{2}-{\sin|\gamma |}^{2}). \end{array}$$

**Figure 3:**
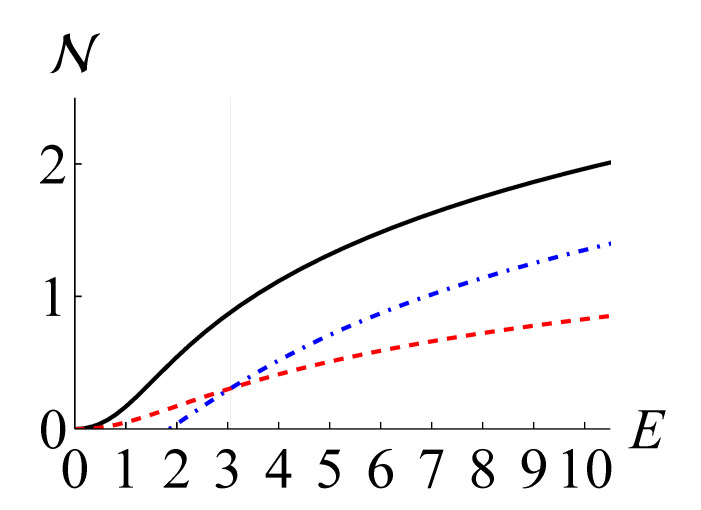
The non-Gaussianity measure based on QRE N(ρ) (black solid), the maximum negentropy of quadrature distributions $N_{\mathrm{KL}}$ (red dashed), and our lower bound $N_{\mathrm{LB}}\left(\rho \right)$ (blue dot-dashed) for a mixture of coherent states against the mean photon number $E=\mathrm{tr}(\rho {\hat{a}}^{†}\hat{a})$.

In Figure 3, we plot N(ρ), $N_{\mathrm{KL}}\left(\rho \right)$, and $N_{\mathrm{LB}}\left(\rho \right)$ as black solid, red dashed, and blue dot-dashed curves, respectively, for the mixture of four coherent states. We observe that $N_{\mathrm{LB}}\left(\rho \right)$ becomes greater than $N_{\mathrm{KL}}\left(\rho \right)$ for E>3.06 (γ>1.75).

### 3.4. Quantum Non-Gaussianity

The mixtures of coherent states are non-Gaussian states but in the convex hull of Gaussian states. In contrast, the Fock states and four-headed cat states are quantum non-Gaussian states, i.e., the states out of the convex hull of Gaussian states. One may ask whether our lower bound $N_{\mathrm{LB}}\left(\rho \right)$ can discriminate quantum non-Gaussian states from classical mixtures of Gaussian states or not. Comparing Figure 2 and Figure 3, it is apparent that $N_{\mathrm{LB}}\left(\rho \right)$ itself cannot serve as a quantum non-Gaussianity witness. However, we want to point out that the dynamics of $N_{\mathrm{LB}}\left(\rho \right)$ under a loss channel can be used for detecting quantum non-Gaussianity. If the following condition is satisfied for a single-mode quantum state ρ, it signifies that ρ is quantum non-Gaussian:

(29) $$N_{\mathrm{LB}}\left(L_{\eta }\circ R\left[\rho \right]\right)-N_{\mathrm{LB}}\left(R\left[\rho \right]\right)<\ln\frac{1+2\eta \bar{n}}{1+2\bar{n}},$$

where R denotes the phase-randomization, $L_{\eta }$ represents the loss channels with the effective transmittance η, and $\bar{n}$ is the mean photon number of ρ. We can derive Equation (29) by reformulating the quantum non-Gaussianity condition in [58]:

(30) $$H_{R[\rho ]}\left(Q\right)<H_{L_{\eta }\circ R\left[\rho \right]}\left(Q\right).$$

Starting from the fact that the reference Gaussian state of R[ρ] is a thermal state with mean photon number $\bar{n}$, it is straightforward to derive $H_{R[\rho_{G}]}\left(Q\right)=\frac{1}{2}\ln\left\{\pi e\left(1+2\bar{n}\right)\right\}$ and $H_{L_{\eta }\circ R\left[\rho_{G}\right]}\left(Q\right)=\frac{1}{2}\ln\left\{\pi e\left(1+2\eta \bar{n}\right)\right\}$, which yields

(31) $$\begin{array}{cc} N_{\mathrm{LB}}\left(L_{\eta }\circ R\left[\rho \right]\right)-N_{\mathrm{LB}}\left(R\left[\rho \right]\right) & =2\left\{H_{L_{\eta }\circ R\left[\rho_{G}\right]}\left(Q\right)-H_{R[\rho_{G}]}\left(Q\right)\right\}-2\left\{H_{L_{\eta }\circ R\left[\rho \right]}\left(Q\right)-H_{R[\rho ]}\left(Q\right)\right\}\\ \; & <2\{H_{L_{\eta }\circ R\left[\rho_{G}\right]}\left(Q\right)-H_{R[\rho_{G}]}\left(Q\right)\}\\ \; & =\ln\frac{1+2\eta \bar{n}}{1+2\bar{n}}. \end{array}$$

It signifies that a sufficiently large decrease in $N_{\mathrm{LB}}\left(R\left[\rho \right]\right)$ under a loss channel is only possible for quantum non-Gaussian states.

## 4. Lower Bounds for Multimode Non-Gaussianity

For a multimode state, quantum state tomography becomes increasingly difficult as the number of modes increases [68]. Thus, it is favorable to estimate the non-Gaussianity of a global quantum state without multimode quantum state tomography.

For an *N*-mode quantum state ρ, the total correlation of the quantum state [76] is given by:

(32) $$T\left[\rho \right]\equiv S(\rho_{1}⊗\cdots ⊗\rho_{N}\left||\rho \right)=\sum_{j=1}^{N}S\left(\rho_{j}\right)-S\left(\rho \right),$$

where $\rho_{j}$ represents the local quantum state of the *j*th mode. If the total correlation of an *N*-mode quantum state ρ has a Gaussian extremality [77] as $T\left[\rho \right]\geq T\left[\rho_{G}\right]$, it allows us to estimate the non-Gaussianity of the global state by measuring the non-Gaussianity of local states as

(33) $$N\left(\rho \right)\geq \sum_{j=1}^{N}N\left(\rho_{j}\right).$$

However, there are counterexamples for the Gaussian extremality, i.e., $T\left[\rho \right]<T\left[\rho_{G}\right]$ [78].

Therefore, we establish two lower bounds for the non-Gaussianity of a multimode quantum state ρ:

(34) $$N\left(\rho \right)\geq L\left(\rho \right)\equiv \max_{j}N\left(\rho_{j}\right),$$

and

(35) $$N\left(\rho \right)\geq \tilde{L}\left(\rho \right)\equiv \sum_{j=1}^{N}N\left({\tilde{\rho }}_{j}\right),$$

where ${\tilde{\rho }}_{i}$ denotes the local quantum state for the *j*th mode of $\tilde{\rho }={\hat{U}}_{S}\rho {\hat{U}}_{S}^{†}$, and ${\hat{U}}_{S}$ represents a symplectic transformation that diagonalizes the covariance matrix of the global quantum state ρ. Note that such a transformation always exists because of the Williamson’s theorem [79].

The first lower bound L(ρ) is a direct consequence of the monotonicity of the non-Gaussianity N(ρ) under a partial trace [69]:

(36) $$N\left(\rho \right)\geq N\left(\rho_{j}\right).$$

The second lower bound $\tilde{L}\left(\rho \right)$ can be derived by using the invariance of the non-Gaussianity under Gaussian unitary operations, i.e., $N\left(\tilde{\rho }\right)=N\left(\rho \right)$, the non-negativity of the total correlation, i.e., T(ρ)≥0, and $T({\tilde{\rho }}_{G})=0$ as

(37) $$N\left(\rho \right)=N\left(\tilde{\rho }\right)\geq \sum_{j=1}^{N}N\left({\tilde{\rho }}_{j}\right).$$

Using Equations (34) and (35) in conjunction with $N_{\mathrm{KL}}\left(\rho \right)$ and $N_{\mathrm{LB}}\left(\rho \right)$, we further establish lower bounds as

(38) $$\begin{array}{cc} L(\rho ) & \geq L_{\mathrm{KL}}\left(\rho \right)\equiv \max_{j}N_{\mathrm{KL}}\left(\rho_{j}\right), \end{array}$$

(39) $$\begin{array}{cc} \tilde{L}\left(\rho \right) & \geq {\tilde{L}}_{\mathrm{KL}}\left(\rho \right)\equiv \sum_{j}N_{\mathrm{KL}}\left({\tilde{\rho }}_{j}\right), \end{array}$$

and

(40) $$\begin{array}{cc} L(\rho ) & \geq L_{\mathrm{LB}}\left(\rho \right)\equiv \max_{j}N_{\mathrm{LB}}\left(\rho_{j}\right), \end{array}$$

(41) $$\begin{array}{cc} \tilde{L}\left(\rho \right) & \geq {\tilde{L}}_{\mathrm{LB}}\left(\rho \right)\equiv \sum_{j}N_{\mathrm{LB}}\left({\tilde{\rho }}_{j}\right). \end{array}$$

These allow us to estimate the non-Gaussianity of a multimode quantum state using quadrature measurements without extensive experimental efforts, i.e., multimode quantum state tomography.

Here, we investigate the CV Werner state [80,81] in the form of $\rho =f|\Xi_{r}\rangle \langle \Xi_{r}{\left|+\left(1-f\right)|0\rangle \langle 0\right|}_{1}⊗{\left|0\rangle \langle 0\right|}_{2}$, where $|\Xi_{r}\rangle =\mathrm{sech}r\sum_{n=0}^{\infty }\tanh^{n}{r|n\rangle }_{1}{|n\rangle }_{2}$ is a two-mode squeezed vacuum with the squeezing parameter *r*. Its covariance matrix is given by

(42) $$\Gamma =\left(\begin{array}{cccc} a & 0 & b & 0\\ 0 & a & 0 & -b\\ b & 0 & a & 0\\ 0 & -b & 0 & a \end{array}\right),$$

where $a=\frac{f}{2}\cosh2r+\frac{1-f}{2}$ and $b=\frac{f}{2}\sinh2r$, which yields

(43) $$N\left(\rho \right)=2g\left(\frac{1}{2}\sqrt{1+4f\left(1-f\right)\sinh^{2}r}\right)-h\left(\frac{1}{2}\left\{1-\sqrt{1-4f\left(1-f\right)\tanh^{2}r}\right\}\right),$$

where h(x)=−xlnx−(1−x)ln(1−x) and

(44) $$\begin{array}{cc} N(\rho_{i})= & g\left(\frac{1}{2}+f\sinh^{2}r\right)+\left(1-f\tanh^{2}r\right)\ln\left(1-f\tanh^{2}r\right)\\ \; & +f\tanh^{2}r\left\{\ln\left(f{\mathrm{sech}}^{2}r\right)+\cosh^{2}r\ln\left(\tanh^{2}r\right)\right\}, \end{array}$$

with i∈{1,2}. Note that the eigenvalues of a classical mixture of two pure states, i.e., ρ=f|Ψ〉〈Ψ|+(1−f)|Φ〉〈Φ|, are given by

(45) $$\lambda_{\pm }=\frac{1}{2}\left\{1\pm \sqrt{1-4f\left(1-f\right)(1-|\langle \Psi |\Phi \rangle {|}^{2})}\right\}.$$

The local quadrature distributions are given by

(46) $$Q_{\rho_{i}}\left(x_{\phi }\right)=fG_{\frac{1}{2}\cosh2r}\left(x_{\phi }\right)+\left(1-f\right)G_{\frac{1}{2}}\left(x_{\phi }\right),$$

where $G_{v}\left(x\right)=\frac{1}{\sqrt{2\pi v}}\exp\left(-\frac{x^{2}}{2v}\right)$ and i∈{1,2}. The covariance matrix can be diagonalized by using a 50:50 beamsplitter. It transforms the CV Werner state into $\tilde{\rho }=f|\xi_{r}\rangle \langle \xi_{r}{|}_{1}⊗|\xi_{r}\rangle \langle \xi_{r}{|}_{2}+{\left(1-f\right)|0\rangle \langle 0|}_{1}⊗{\left|0\rangle \langle 0\right|}_{2}$, where $|\xi_{r}\rangle =\sqrt{\mathrm{sech}r}\sum_{n=0}^{\infty }{\left(-\tanh r\right)}^{n}\frac{\sqrt{(2n)!}}{2^{n}n!}|2n\rangle$ is a single-mode squeezed vacuum with the squeezing parameter *r*. The covariance matrix of the transformed state $\tilde{\rho }$ becomes diag(a−b,a+b,a−b,a+b) which yields

(47) $$N\left({\tilde{\rho }}_{i}\right)=g\left(\frac{1}{2}\sqrt{1+4f\left(1-f\right)\sinh^{2}r}\right)-h\left(\frac{1}{2}\left\{1-\sqrt{1-4f(1-f)(1-\mathrm{sech}r)}\right\}\right),$$

with i∈{1,2}. The local quadrature distributions are given by

(48) $$Q_{{\tilde{\rho }}_{i}}\left(x_{\phi }\right)=fG_{v_{\phi }}\left(x_{\phi }\right)+\left(1-f\right)G_{\frac{1}{2}}\left(x_{\phi }\right),$$

where $v_{\phi }=\frac{1}{2}\left(e^{-2r}\cos^{2}\phi +e^{2r}\sin^{2}\phi \right)$ and i∈{1,2}.

**Figure 4:**
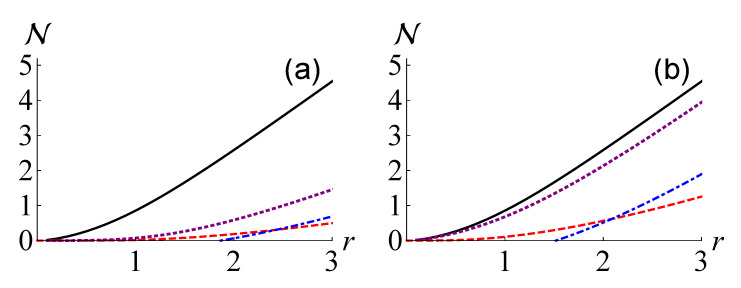
The non-Gaussianity measure based on QRE N(ρ) (black solid curve) and its lower bounds for the CV Werner state with $f=\frac{1}{2}$ against the squeezing parameter *r*. We examine the cases without and with the help of the Gaussian unitary operation in (**a**,**b**), respectively. The lower bounds based on local non-Gaussianity measures, i.e., L(ρ) for (**a**) and $\tilde{L}\left(\rho \right)$ for (**b**), the maximum negentropy of quadrature distributions, i.e., $L_{\mathrm{KL}}\left(\rho \right)\equiv \max_{j}N_{\mathrm{KL}}\left(\rho_{j}\right)$ for (**a**) and ${\tilde{L}}_{\mathrm{KL}}\left(\rho \right)=\sum_{j}N_{\mathrm{KL}}\left({\tilde{\rho }}_{j}\right)$ for (**b**), and our lower bound, i.e., $L_{\mathrm{LB}}\left(\rho \right)\equiv \max_{j}N_{\mathrm{LB}}\left(\rho_{j}\right)$ for (**a**) and ${\tilde{L}}_{\mathrm{LB}}\left(\rho \right)=\sum_{j}N_{\mathrm{LB}}\left({\tilde{\rho }}_{j}\right)$ for (**b**), are plotted as purple dotted, red dashed, and blue dot-dashed curves, respectively.

For the CV Werner states, we investigated the performance of the estimation methods without and with the help of a Gaussian unitary operation, as shown in Figure 4a,b, respectively. It is observed that $L_{\mathrm{LB}}\left(\rho \right)>L_{\mathrm{KL}}\left(\rho \right)$ and ${\tilde{L}}_{\mathrm{LB}}\left(\rho \right)>{\tilde{L}}_{\mathrm{KL}}\left(\rho \right)$ occur when r>2.43 and r>2.08, respectively. In addition, the results clearly show that a Gaussian unitary operation can significantly increase the values of the lower bounds.

## 5. Application in Entanglement Detection

Here, we explore how our lower bound can be used to detect quantum entanglement. Following Refs. [44,46], we first reformulate Equation (4) as follows:

(49) $$\sqrt{\det\Gamma_{\rho }}=g^{-1}\left(S\left(\rho_{G}\right)\right)=g^{-1}\left(N\left(\rho \right)+S\left(\rho \right)\right).$$

As $g^{-1}\left(x\right)$ is a monotonically increasing function of *x*, $N\left(\rho \right)\geq N_{\mathrm{LB}}\left(\rho \right)$, and S(ρ)≥0, we have

(50) $$\sqrt{\det\Gamma_{\rho }}\geq g^{-1}\left(\max\left[0,N_{\mathrm{LB}}\left(\rho \right)\right]\right),$$

where we take the maximum between zero and $N_{\mathrm{LB}}\left(\rho \right)$ by considering that $N_{\mathrm{LB}}\left(\rho \right)$ can be negative. Note that Equation (50) is an improved version of the Robertson–Schrödinger (RS) uncertainty relation, i.e., $\sqrt{\det\Gamma_{\rho }}\geq \frac{1}{2}$:

(51) $$\sqrt{\det\Gamma_{\rho }}\geq g^{-1}\left(\max\left[0,N_{\mathrm{LB}}\left(\rho \right)\right]\right)\geq \frac{1}{2}.$$

We now explain how Equation (50) can be used for entanglement detection. First, we apply partial transposition to a multimode quantum state ρ. The partially transposed state $\rho^{\mathrm{PT}}$ remains as a legitimate quantum state if ρ is separable. Therefore, if the partially transposed state $\rho^{\mathrm{PT}}$ fails to be a legitimate quantum state, then it witnesses that ρ is entangled. If we know the density matrix of ρ, it is straightforward to test the legitimacy of $\rho^{\mathrm{PT}}$. A negative eigenvalue of $\rho^{\mathrm{PT}}$ is enough to reveal that ρ is entangled. However, we need to perform resource-intensive quantum state tomography to obtain the complete information on ρ. Here, we are interested in resource-efficient certification of entanglement using uncertainty relations. After partial transposition, we apply a symplectic transformation ${\hat{U}}_{S}$ to $\rho^{\mathrm{PT}}$ for diagonalizing the covariance matrix of $\rho^{\mathrm{PT}}$. Following Williamson’s theorem [79], such a transformation always exists. If there is a local mode of $\bar{\rho }={\hat{U}}_{S}\rho^{\mathrm{PT}}{\hat{U}}_{S}^{†}$ violates the RS uncertainty relation, i.e., $\sqrt{\det\Gamma_{{\bar{\rho }}_{j}}}<\frac{1}{2}$, it shows that the entanglement of ρ is detectable by the Gaussian PPT criteria. Here, we employ Equation (50) instead of the RS uncertainty relation to detect non-Gaussian entanglement beyond the Gaussian PPT criteria. If there is a local mode ${\bar{\rho }}_{j}$ fulfills the following condition,

(52) $$\sqrt{\det\Gamma_{{\bar{\rho }}_{j}}}<g^{-1}\left(\max\left[0,N_{\mathrm{LB}}\left({\bar{\rho }}_{j}\right)\right]\right),$$

it certifies the entanglement of ρ.

Before going further, let us describe a standard procedure to test Equation (52) experimentally. We first determine the covariance matrix $\Gamma_{\rho }$ of a multi-mode quantum state ρ by homodyne detection [82]. As partial transposition on *j*th mode only flips the sign of the *j*th momentum quadrature, we can deduce the covariance matrix $\Gamma_{\rho^{\mathrm{PT}}}$ of the partially transposed state $\rho^{\mathrm{PT}}$ simply changing the sign of the relevant covariance matrix elements [83]. For instance, if we apply partial transposition on the second mode of a two-mode quantum state ρ whose covariance matrix is given by

(53) $$\Gamma_{\rho }=\left(\begin{array}{cccc} \Gamma_{11} & \Gamma_{12} & \Gamma_{13} & \Gamma_{14}\\ \Gamma_{21} & \Gamma_{22} & \Gamma_{23} & \Gamma_{24}\\ \Gamma_{31} & \Gamma_{32} & \Gamma_{33} & \Gamma_{34}\\ \Gamma_{41} & \Gamma_{42} & \Gamma_{43} & \Gamma_{44} \end{array}\right),$$

we obtain the covariance matrix of the partially transposed state $\rho^{\mathrm{PT}}$ as

(54) $$\Gamma_{\rho^{\mathrm{PT}}}=\left(\begin{array}{cccc} \Gamma_{11} & \Gamma_{12} & \Gamma_{13} & -\Gamma_{14}\\ \Gamma_{21} & \Gamma_{22} & \Gamma_{23} & -\Gamma_{24}\\ \Gamma_{31} & \Gamma_{32} & \Gamma_{33} & -\Gamma_{34}\\ -\Gamma_{41} & -\Gamma_{42} & -\Gamma_{43} & \Gamma_{44} \end{array}\right).$$

We, then, determine the symplectic transformation ${\hat{U}}_{S}$ that diagonalizes the covariance matrix $\Gamma_{\rho^{\mathrm{PT}}}$ by using the algorithm in [84]. Examining the diagonalized covariance matrix, we can calculate $\sqrt{\det\Gamma_{{\bar{\rho }}_{j}}}$, i.e., the left-hand side of Equation (52). The symplectic transformation ${\hat{U}}_{S}$ is a Gaussian unitary operation directly related to a linear transformation of quadrature operators as ${\hat{U}}_{S}^{†}\hat{q_{j,\phi }}{\hat{U}}_{S}=\sum_{k=1}^{N}c_{jk}{\hat{q}}_{k,\phi_{k}}$ [2]. We assume that partial transposition is applied on the last mode without loss of generality. Then, we have

(55) $$\begin{array}{cc} \mathrm{tr}[\bar{\rho }{\hat{q}}_{j,\phi }] & =\mathrm{tr}[\rho^{\mathrm{PT}}{\hat{U}}_{S}^{†}\hat{q_{j,\phi }}{\hat{U}}_{S}]\\ \; & =\mathrm{tr}[\rho^{\mathrm{PT}}\sum_{k=1}^{N}c_{jk}{\hat{q}}_{k,\phi_{k}}]\\ \; & =\mathrm{tr}[\rho \left(\sum_{k=1}^{N-1}c_{jk}{\hat{q}}_{k,\phi_{k}}+c_{jN}{\hat{q}}_{N,-\phi_{N}}\right)], \end{array}$$

which indicates that we can measure the quadrature distributions for $N_{\mathrm{LB}}\left({\bar{\rho }}_{j}\right)$, i.e., the right-hand side of Equation (52), by using an adequately chosen Gaussian unitary operation and homodyne detection. For instance, if the covariance matrix of a two-mode quantum state has a symmetry, such as Equation (42), ${\hat{U}}_{S}$ becomes a 50:50 beam-splitting operation. In this case, we can obtain $N_{\mathrm{LB}}\left({\bar{\rho }}_{1}\right)$ by measuring the quadrature distributions for ${\hat{Q}}_{+}=\frac{1}{\sqrt{2}}\left({\hat{q}}_{1}+{\hat{q}}_{2}\right)$ and ${\hat{P}}_{-}=\frac{1}{\sqrt{2}}\left({\hat{p}}_{1}-{\hat{p}}_{2}\right)$.

Here, we investigate the CV Werner states in the form of $\rho =f|\Xi_{r}\rangle \langle \Xi_{r}|+\left(1-f\right)\tau_{\bar{n},1}⊗\tau_{\bar{n},2}$. Without loss of generality, we assume that the squeezing parameter *r* is positive. By applying partial transposition and a 50:50 beam-splitting operation, we have $\bar{\rho }={\hat{U}}_{\mathrm{BS}}\rho^{\mathrm{PT}}{\hat{U}}_{\mathrm{BS}}^{†}=f\tau_{{\bar{n}}_{-},1}⊗\tau_{{\bar{n}}_{+},2}+\left(1-f\right)\tau_{\bar{n},1}⊗\tau_{\bar{n},2}$ with ${\bar{n}}_{\pm }=\pm e^{\pm r}\sinh r$. Note that ${\bar{n}}_{+}$ and ${\bar{n}}_{-}$ are positive and negative, respectively, for r>0. Although ${\bar{\rho }}_{2}=f\tau_{{\bar{n}}_{+}}+\left(1-f\right)\tau_{\bar{n}}$ is always physical, ${\bar{\rho }}_{1}=f\tau_{{\bar{n}}_{-}}+\left(1-f\right)\tau_{\bar{n}}$ can be unphysical. The negativity in the photon number distribution of ${\bar{\rho }}_{1}$ exhibits the entanglement of the CV Werner state. For instance, ${\bar{\rho }}_{1}$ with $f=\frac{1}{2}$ and $\bar{n}=1$ becomes unphysical for r>0.21.

**Figure 5:**
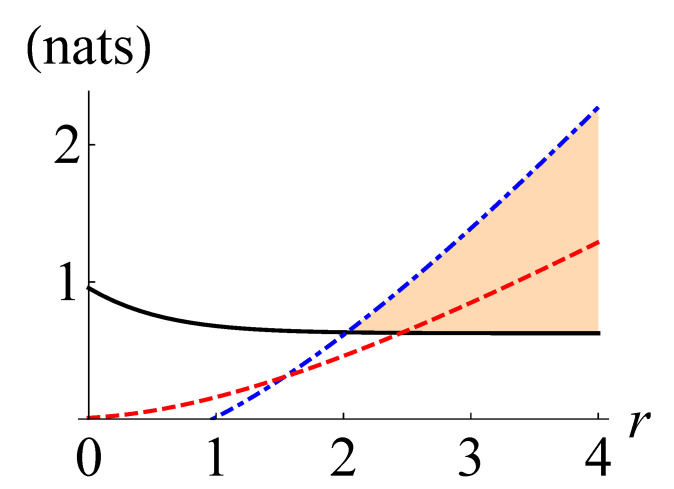
The entropic quantities $g(\sqrt{\det\Gamma_{{\bar{\rho }}_{1}}})$, $N_{\mathrm{KL}}\left({\bar{\rho }}_{1}\right)$, and $N_{\mathrm{LB}}\left({\bar{\rho }}_{1}\right)$ for a CV Werner state $\rho =f|\Xi_{r}\rangle \langle \Xi_{r}|+\left(1-f\right)\tau_{\bar{n},1}⊗\tau_{\bar{n},2}$ with $f=\frac{1}{2}$ and $\bar{n}=1$ are plotted with respect to the squeezing parameter *r* as black solid, red dashed, and blue dot-dashed curves, respectively. The shaded region indicates that Equation (52) reveals the quantum entanglement of the CV Werner state ρ, which is undetectable by using the Gaussian PPT criteria.

In Figure 5, we plot $g(\sqrt{\det\Gamma_{{\bar{\rho }}_{1}}})$, $N_{\mathrm{KL}}\left({\bar{\rho }}_{1}\right)$, and $N_{\mathrm{LB}}\left({\bar{\rho }}_{1}\right)$ as black solid, red dashed, and blue dot-dashed curves, respectively, for the CV Werner states with $f=\frac{1}{2}$ and $\bar{n}=1$ whose entanglement is undetectable by the Gaussian PPT entanglement criteria. It is noteworthy that $g\left(\sqrt{\det\Gamma_{{\bar{\rho }}_{1}}}\right)<N_{\mathrm{KL}}\left({\bar{\rho }}_{1}\right)$ is the entanglement condition derived in [46]. We observe that $N_{\mathrm{KL}}\left({\bar{\rho }}_{1}\right)$ and $N_{\mathrm{LB}}\left({\bar{\rho }}_{1}\right)$ allow the detection of the entanglement of the CV Werner states when r>2.45 and r>2.03, respectively. The results indicate that our method can detect non-Gaussian entangled states that cannot be detected by the method proposed in [46].

## 6. Concluding Remarks

We derived observable lower bounds for a non-Gaussianity measure based on QRE. We first established a lower bound for a single-mode quantum state as a function of the negentropies of quadrature distributions with the maximum and minimum variances, and we showed that it could perform better than the previously proposed bound in [46]. We also formulated the strategies for estimating the QRE-based non-Gaussianity of a multimode quantum state using local quantities with or without leveraging a Gaussian unitary operation. Furthermore, we explored how our lower bound could be employed to detect non-Gaussian entanglement beyond the Gaussian PPT entanglement criteria.

We hope that our contributions will facilitate efficient and experimentally friendly certification methods for CV quantum resources. Although we here employed the quadrature distributions to address the non-Gaussianity of quantum states, there also exist other forms of probability representation for quantum states [85]. It will be intriguing to find a quantitative relation between the non-Gaussianity of the tomographic probability distributions and other non-Gaussianity measures. In addition, it will be worthwhile to extend our estimation method to more elaborate measures, such as the non-Gaussianity measure for quantum-state correlation [78] and the measures for quantum non-Gaussianity [48,49,50,51,52], i.e., a more robust form of non-Gaussianity. This topic will be investigated in future research.

## Abbreviations

The following abbreviations are used in this manuscript:

CV	Continuous-Variable
QRE	Quantum Relative Entropy
KLD	Kullback–Leibler Divergence
